# ”Unmasking the Villain”: Integrative Taxonomy Reveals the Real Identity of the Key Pest (Thysanoptera: Thripidae) of Peanuts (*Arachis hypogaea* L.) in South America †

**DOI:** 10.3390/insects13020120

**Published:** 2022-01-24

**Authors:** Élison Fabrício Bezerra Lima, Áquila Rayane Silva de Alencar, Frederico Nanini, Marcos Doniseti Michelotto, Alberto Soares Corrêa

**Affiliations:** 1 Coleção de História Natural da UFPI, Campus Amílcar Ferreira Sobral, Universidade Federal do Piauí, BR 343, Km 3.5, Floriano 64808-605, Brazil; aquilaalencar@outlook.com; 2 Departamento de Entomologia e Acarologia, Escola Superior de Agricultura Luiz de Queiroz, Universidade de São Paulo, Avenida Pádua Dias, 11, Piracicaba 13418-900, Brazil; frederico.nanini.santos@usp.br (F.N.); ascorrea@usp.br (A.S.C.); 3 Agência Paulista de Tecnologia dos Agronegócios, Polo Regional Centro Norte, Rodovia Washington Luis, Km 372, C. Postal 24, Pindorama 15830-000, Brazil; michelotto@apta.sp.gov.br

**Keywords:** DNA barcoding, new species, pest management

## Abstract

**Simple Summary:**

In this work, we aimed to resolve the identification of the peanut thrips, the key pest of *Arachis hypogaea* in South America. Based on morphological, biological, and molecular data, we conclude that the name historically applied to this pest, *Enneothrips flavens*, constitutes a misidentification and that the peanut thrips is actually an undescribed species, *Enneothrips enigmaticus* sp. n.

**Abstract:**

The peanut thrips, *Enneothrips enigmaticus* sp. n., is the key pest of *Arachis hypogaea* L. in South America, where it can cause yield losses of up to 85%. This species has historically been identified as *Enneothrips flavens*, but access to the holotype of this species and freshly collected material from southeastern and northern Brazil revealed that specimens commonly collected on peanut crops are not conspecific with *E. flavens*. Biological, molecular, and morphological assessments were carried out and led to the conclusion that the key pest of *A. hypogaea* belongs to a previously undescribed species: *Enneothrips enigmaticus* sp. n.

## 1. Introduction

The peanut thrips is the key pest of peanuts crops, *Arachis hypogaea* L. (Fabaceae), in South America. This pest is found in Argentina, Brazil, and Paraguay and can cause losses of up to 85% [1,2]. The species is commonly found on leaflets, where it feeds on the tissue, causing bronze markings and deformation [1,3].

The first record of a name applied to the peanut thrips was *Frankliniella fusca* (Hinds), which was a misidentification subsequently corrected by Gallego de Sureda, who identified the species as *Enneothrips* (*Enneothripiella*) *flavens* Moulton [4]. This name was previously proposed by Moulton [5] to describe a species based on a unique female specimen collected in Minas Gerais state, Brazil, on “Indian tea” plants. Due to the identification by Gallego de Sureda [4], peanut thrips has been referred to as *E. flavens* and host-specific of *A. hypogaea* [6].

However, specimens of *Enneothrips* recently collected on plants other than *A. hypogaea* were identified as *E. flavens*. This fact suggests that either *E. flavens* is not host-specific or *Enneothrips* specimens previously collected from *A. hypogaea* were misidentified. Both hypotheses have a serious impact because if the species is not host-specific to *A. hypogaea*, the control tactics must take into account that alternative hosts may harbor the pest when peanut is not cultivated. On the other hand, if the name of the peanut thrips is not being properly used, the scientific nomenclature of all the literature on this species is incorrect. Thus, this taxonomic issue about the peanut thrips has not only systematic interest for a species, but also an economic impact.

Here, we report biological, molecular, and morphological evidence that reveals that the name currently applied to the peanut thrips is not appropriate. Therefore, we “unmask the villain” and reveal the real identity of this important pest species by proposing its reclassification as a new species to science—*Enneothrips enigmaticus* sp. n.

## 2. Materials and Methods

Fresh material of the peanut thrips was manually collected from this crop in Rio Branco (9°55′43.24″ S; 67°53′54.80″ W), Acre, and Jaboticabal (21°14′23.40″ S; 48°17′45.22″ W), São Paulo, Brazil. In addition, specimens to which the name *Enneothrips flavens* could be applied, were collected from fabaceous herbs and *Adenanthera macrocarpa* in Pedralva (22°15′55.00″ S; 45°24′37.00″ W), Minas Gerais, and from *Campomanesia guazumifolia* in Santo Antonio do Pinhal (22°48′38.55″ S; 45°42′14.24″ W), São Paulo, close to southern Minas Gerais, the type locality of the species. All of them were stored in ethanol 100% for slide mounting and DNA extraction. In addition, *Enneothrips* spp. specimens deposited in several collections were examined. Collection details are available in material examined. To correct the identification and description of the *Enneothrips* spp. collected on *A. hypogaea* and other hosts, we performed biological, morphological, and molecular assessments. In addition, we modelled the potential distribution of the new species based on 19 climactic factors.

### 2.1. Biological Assessment

Whenever possible, plant species with *Enneothrips* spp. were identified and, when available, larvae were collected to establish true host associations. Data available in [6] were used as evidence for host-specificity of the peanut thrips to *A. hypogaea*. As a consequence, second-instar larvae of both species discussed herein are described.

### 2.2. Morphological Assessment

As for morphological studies, comparison of the collected material with the holotype of *E. flavens* was carried out. In addition, the specimens were compared with type material of all other four valid *Enneothrips* species and with additional specimens whose morphology matches with *E. flavens* holotype in CHNUFPI, ESALQ, and USNM. Fresh material was prepared onto permanent microscope slides based on the technique proposed in [7] (adults) and in [8] (immatures). Photomicrographs of the specimens were taken under a Zeiss Axio Lab A1 microscope (Carl Zeiss Microscopy, Jena, Germany) with phase contrast and a Zeiss Axiocam ERc5 camera (Carl Zeiss Microscopy, Jena, Germany) attached.

In addition, SEM (Scanning Electron Microscopy) images were taken for specimens collected from peanuts on a Hitachi TableTop Scanning Microscope TM3000 (Hitachi High-Technologies Corporation, Tokyo, Japan) following the manufacturer’s guidelines.

Depositaries acronyms mentioned in this text are: CHNUFPI (Coleção de História Natural da Universidade Federal do Piauí, Floriano, Brazil), ESALQ (Escola Superior de Agricultura “Luiz de Queiroz”, Universidade de São Paulo, Piracicaba, Brazil), MLP (Museo de La Plata, La Plata, Argentina), CAS (California Academy of Sciences, San Francisco, CA, USA), and USNM (Smithsonian’s National Museum of Natural History, Washington, DC, USA).

### 2.3. Molecular Assessment

#### 2.3.1. gDNA Extraction

Total genomic DNA was extracted from two individuals of *E. flavens* from Santo Antonio do Pinhal, SP, Brazil, and seven individuals of *Enneothrips* from peanuts from Jaboticabal (n = 4) and Rio Branco (n = 3), previously conserved in ethanol 100%. Non-destructive DNA extraction was performed with a modified protocol from [9]. Initially, insects were individualized in 1.5 mL Eppendorf tubes and were immersed in 0.3 mL of digestion buffer [3-mM CaCl_2_, 2% sodium dodecyl sulphate (SDS), 40 mM dithiothreitol (DTT), 100 mM Tris buffer pH 8, and 100 mM NaCl] with addition of 12.5 µL of proteinase K (20 µg ml^−1^) and incubated at 65 °C overnight (≈16 h). At the end of the incubation, the insects were removed and stored in 100% ethanol at −20 °C for morphological identification. After insect removal, the digestion buffer was mixed with 0.3 mL of chloroform + isoamyl alcohol (24:1) and centrifuged at 14,000 rpm for 20 min. The supernatant solution was transferred to a new Eppendorf with 30 µL of sodium acetate (3.0 M, pH 5.2), 2.5 µL of glycogen (5 mg ml^−1^), and 0.235 mL of iced 100% isopropanol. The mixture was gently vortexed and placed overnight at −20 °C and subsequently centrifuged at 14,000 rpm at 4 °C for 30 min. The liquid was removed, and the DNA pellet was serially washed with 0.4 mL of 70% ethanol and 95% ethanol. Lastly, the DNA pellet was air-dried, suspended in 25 µL of MilliQ-H_2_O, and stocked in a −20 °C freezer.

#### 2.3.2. COI Amplification and Sequencing

The cytochrome c oxidase subunit I (*COI*) gene fragment was amplified by polymerase chain reaction (PCR) using the universal primers LCO1490 (5′ GGTCAACAAATCATAAAGATATTGG 3′) and HCO2198 (5′ TAAACTTCAGGGTGACCAAAAAATCA 3′) [10]. The PCR was performed in a total of 25 µL containing 3 µL genomic DNA (gDNA), 18.25 µL MilliQ-H_2_O, 0.25 µL 10X PCR Buffer Mg^2+^-free (Thermo Fisher Scientific ™), 1.25 µL MgCl_2_ (50 mM) (Thermo Fisher Scientific ™, Carlsbad, CA, USA), 0.125 µL dNTP (10 mM) (Sinapse Inc^®^) (Hollywood, FL, USA), 1 µL of each primer (5 µM), and 0.125 µL Platinum^®^ (Carlsbad, CA, USA) Taq DNA Polymerase (5 U µL^−1^) (Thermo Fisher Scientific ™) (Carlsbad, CA, USA). The PCR amplification conditions used were 94 °C for 3 min for primary denaturation, then 35 cycles at 94 °C for 30 s, 53 °C for 45 s, 72 °C for 2 min, with a final extension at 72 °C for 10 min. PCR amplicons were visualized under ultraviolet light after electrophoresis using 3 μL amplicon in 2% (w v^–1^) agarose gel stained with SYBR Safe (Life Technologies). The subsequent purification process was performed using 1 µL (20 U µL^−1^) of Exonuclease I (Thermo Fisher Scientific ™) and 2 µL (1 U µL^−1^) of FastAP ™ Thermosensitive Alkaline Phosphatase (Thermo Fisher Scientific ™) for 10 µL of PCR final product. The conditions of thermocycler used for purification involved 30 min at 37 °C, followed by 15 min at 80 °C. The bidirectional sequencing was performed by the Sanger method using the same PCR primers in the Animal Biotechnology Laboratory at ESALQ, University of São Paulo.

### 2.4. Molecular Data Analysis

The chromatogram of each COI sequence was verified, aligned, and edited in a consensus sequence using Sequencher 4.8 (Gene Codes Corp., Ann Arbor, MI). Afterwards, COI sequences were aligned, and the presence of nuclear paralogs of mitochondrial origin (NumtS) [11] was observed following steps described in [12]: (i) insertions/deletions (indels), (ii) stop codons leading to premature protein termination, and (iii) increased rates of non-synonymous mutations. The presence of signatures (i) and (ii) would be enough to consider a sequence as a NumtS. The presence of signature (iii) would be used to confirm the NumtS status of the sequence. The genetic distance among haplotypes was estimated using the Kimura-two-parameters (K2P) model with 5000 bootstrap replications in MEGA X [13]. A Bayesian phylogenetic tree was estimated using the best substitution model of evolution GTR+I selected using the software MRMODELTEST v2.3 [14]. The Bayesian phylogenetic analyses were carried out in MRBAYES v3.1.2 [15], using two simultaneous runs of 25 million generations with one cold and three heated chains in each run. At the end of the runs, the first 25% of the trees were discarded as burn-in samples. The consensus tree of the two independent runs was obtained with posterior probabilities > 0.50. The COI sequence of *Frankliniella occidentalis* (NCBI accession number: HQ930545) was used as outgroup. No sequences of other *Enneothrips* spp. are available in NCBI; thus, sequences of the common species *F. occidentalis* were chosen exclusively to estimate the genetic distance, not phylogenetic inferences.

### 2.5. Species Distribution Modelling

As the distribution of *E. enigmaticus* sp. n. is not well studied, the potential distribution in the Americas was modelled based on taxonomic validated species records on peanuts from Thysanoptera collections from USNM, CHNUFPI MLP, and ESALQ (Table 1). For non-georeferenced records, we used the label information to retrieve the most probable coordinates. A total of 19 environmental variables were used to estimate the potential distribution, which included precipitation, temperature, moisture index, and radiation, at a 10 min spatial resolution. These were obtained from the WorldClim dataset and cover climate information in the period 1970–2000. Analysis for *E. flavens* was not performed because there are very few records of this species to make the analysis possible.

Modelling was performed through multivariate analysis and maximum entropy using MaxEnt 3.4.4 [16]. MaxEnt estimates the probability of occurrence based on environmental parameters and has predictive power even with small datasets [17]. Model settings were defined after a bias analysis using the software R 4.0.2. The chosen model, with the least delta AIC, included linear features with a regularization multiplier of 1.0.

This information is relevant not only because of the economic importance of *E. enigmaticus* sp. n., but also because the model results provide indication of a possible shared distribution with *E. flavens*, which can help understand the evolutionary pathways of both species.

## 3. Results

**Figure 1:**
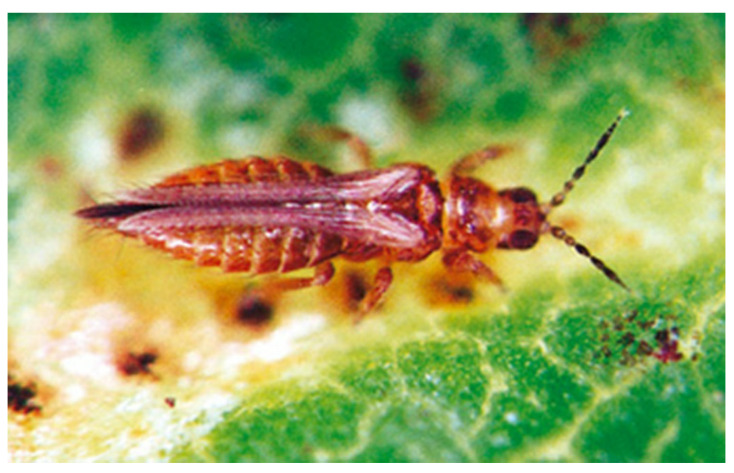
*Enneothrips enigmaticus* sp. n., female, on peanut (*Arachis hypogaea*) (Photo: ESALQ Entomological Museum).

Based on morphological, biological and molecular evidence detailed below, we describe the new species *E. enigmaticus* sp. n. as the pest on peanuts (Figure 1). We re-evaluate the morphological description of *E. flavens* while also describing the adult female and male and larvae II of both species. This information is essential for the recognition of both species and constitutes the first step towards the adoption of management tactics for the key peanut pest.

*Enneothrips enigmaticus* sp. n.
http://zoobank.org/urn:lsid:zoobank.org:act:E2CBCE8C-7508-4C46-B380-5AF2602FFE9D

**Figure 2:**
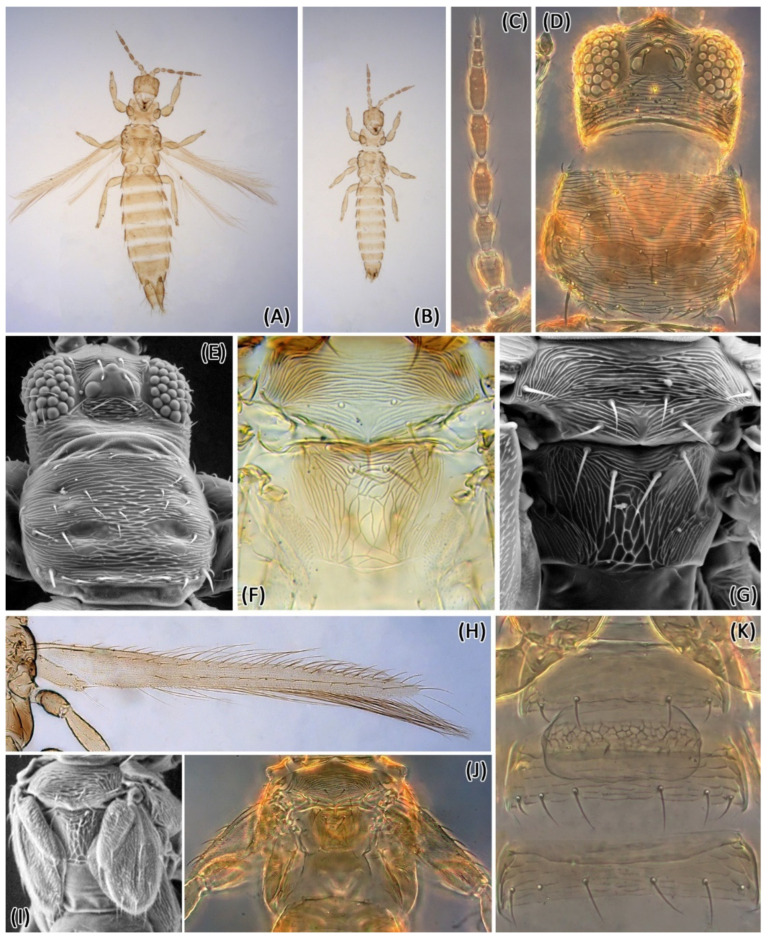
*Enneothrips enigmaticus* sp. n. (**A**) Female; (**B**) Male; (**C**) Antenna; (**D**) Head and pronotum; (**E**) Head and pronotum (SEM); (**F**) Meso- and metanotum; (**G**) Meso- and metanotum (SEM); (**H**) Fore wing (female); (**I**) Wings (male) (SEM); (**J**) Wings (male); (**K**) Abdominal sternites II–IV (male).

*Female macroptera*. Color orange to reddish brown (Figure 2A). Antennal segments I and II light brown, III brown with base pale, IV and V brown with extreme base pale, VI–IX brown (Figure 2C). Head, thorax, and abdomen reddish brown, somewhat darker laterally (Figure 2D,F); fore wing reddish brown with pale basal fifth (Figure 2H); legs yellow; abdominal tergites uniformly reddish brown. Head with ocellar area without sculpture; ocellar setae III within the ocellar triangle (Figure 2D,E); pronotum and mesonotum transversely striate, around 20 lines of sculpture on mesonotum (Figure 2F,G); metanotum longitudinally sculptured on lateral thirds, transversely sculptured on anterior third, and reticulate on the median two-thirds, without internal lines inside reticles (Figure 2F,G). Abdominal tergites transversely striate laterally, about 15 lines of sculpture; tergite VIII with posteromarginal comb complete. Abdominal sternites II–VIII with microtrichia medially; posteromarginal setae anterior to margin.

Measurements (holotype female in microns): Body length 1375. Head length 85; width 130; ocellar setae III length 15. Pronotum length 105; width 150; posteroangular setae length 35. Fore wing length 600. Antennal segments I–IX length 18, 32, 38, 42, 39, 39, 12, 10, 15.

*Male brachyptera*. Similar to female (Figure 2B), but smaller, brachypterous (Figure 2I,J), with a broad pore plate between the abdominal sternites II and III (Figure 2K).

Measurements (paratype male in microns): Body length 1000. Head length 65; width 110; ocellar setae III, length 12. Pronotum length 90; width 125; posteroangular setae length 30. Fore wing length 100. Pore plate length 30; width 78. Antennal segments I–IX length 12, 30, 30, 38, 32, 38, 10, 8, 10.

**Figure 3:**
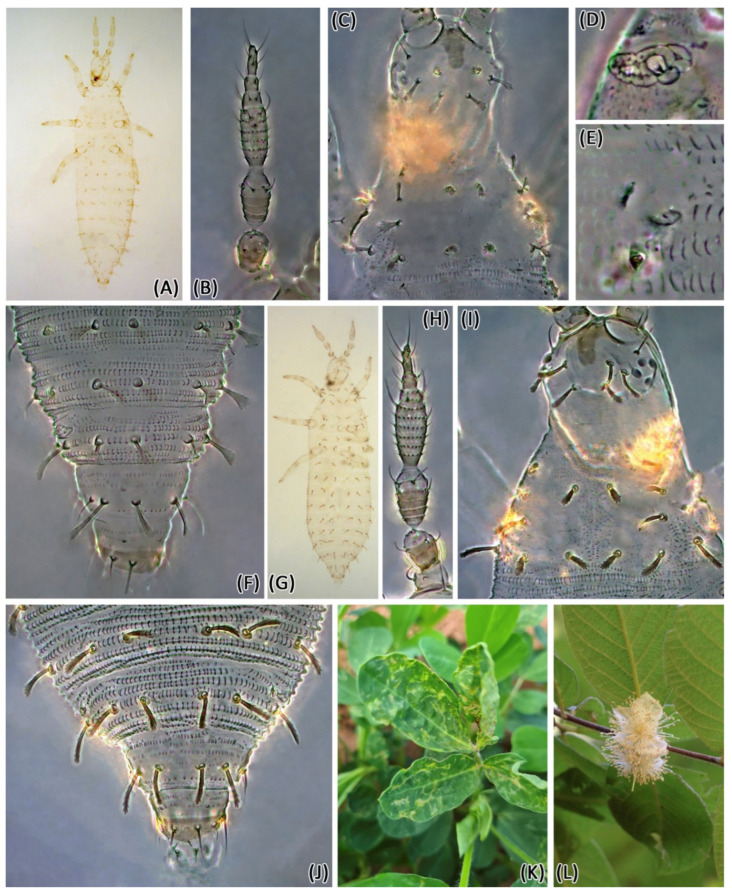
*Enneothrips* spp. larvae II and hosts. *Enneothrips enigmaticus* sp. n.: (**A**) Habitus; (**B**) Antenna; (**C**) Head and pronotum; (**D**) Mesothoracic spiracle; (**E**) Abdominal tergite II spiracle; (**F**) Abdominal tergites VI-X. *Enneothrips flavens*: (**G**) Habitus; (**H**) Antenna; (**I**) Head and thorax; (**J**) Abdominal tergites. (**K**) Damage of *E. enigmaticus* sp. n. on peanut leaflets; (**L**) *Campomanesia guazumifolia*, host of *E. flavens*.

*Larva II*. Color, pale (Figure 3A) with brown areas anteromedially on head (Figure 3C), posteriorly on abdominal segment X (Figure 3F) and on the bases of femora and tibiae. Antennal segment I pale, II brown with pale apex, III brown with pale base and apex, IV–VII brown. Antennal segment IV with rows of microtrichia (Figure 3B). Body dorsal setae expanded, except for cephalic pair D3 acute (Figure 3C). Ventral setae acute. Body densely covered with sclerotized plates, absent on head, less numerous on pronotum and abdominal tergites IX and X (Figure 3F). Spiracles on mesonotum and abdominal tergites II and VIII; spiracle facets variably with zero or one pore (Figure 3D,E). Abdominal tergites IX and X with one pair of campaniform sensilla.

Measurements (in microns). Body length 1200. Head length 90; width 80; D1 setae length 13; D4 setae length 15. Pronotum length 85; width 150; D5 setae length 15; D6 setae length 22. Abdominal tergites IX length 60; width 75; setae D1 length 25; setae D2 length 30; X length 35; width 50. Antennal segments I–VII length 12, 27, 40, 42, 8, 10, 18.

Material studied. Holotype female. Brazil. São Paulo: Jaboticabal, UNESP-FCAV, *Arachis hypogaea*, 12.vii.2016 (J.R. Lima col.) (CHNUFPI).

Paratypes. BRAZIL. Same data as holotype, 4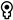 4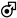 4 larvae II (CHNUFPI). São Paulo: Campinas, same host, 18.i.1994, 4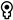 (D. Gabriel col.) (ESALQ); Pindorama, same host, 15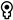 2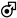 xii.2016 (M. Michelotto col.) (CHNUFPI). Acre: Rio Branco, Embrapa Acre, *Arachis pintoi*, 17.vii.2014, 1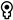 (R.S. Santos col.); same locality and host, 27.vii.2019 1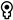 (E.F.B. Lima col.) (CHNUFPI).

Non-type material. BRAZIL. São Paulo: Campinas, *Arachis hypogaea*, 14.xii.1993, 3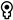; 21.xii.1993, 19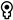; 4.i.1994, 49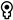 2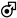 (D. Gabriel col.); Piracicaba, same host, 10.iv.1991, 12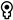 2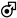 (R.C. Monteiro col.); Tupi Paulista, same host, no dates, 1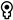 (Adalberto col.); Jaboticabal, same host, 1995, 2 larvae II (M.G.A. Lima col.) (ESALQ); Santa Adélia, same host, iii.2014, 2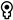; Itápolis, same host, iii.2014, 1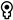 (M. Michelotto col.) (CHNUFPI). Minas Gerais: Uberlândia, 30.vi.2020, 1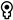 (G. Berchieri col.) (CHNUFPI) PARAGUAY. Boquerón, same host, 3.ii.2012, 2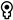 (L.R.G Segnana col.) (CHNUFPI).

*Enneothrips flavens*

**Figure 4:**
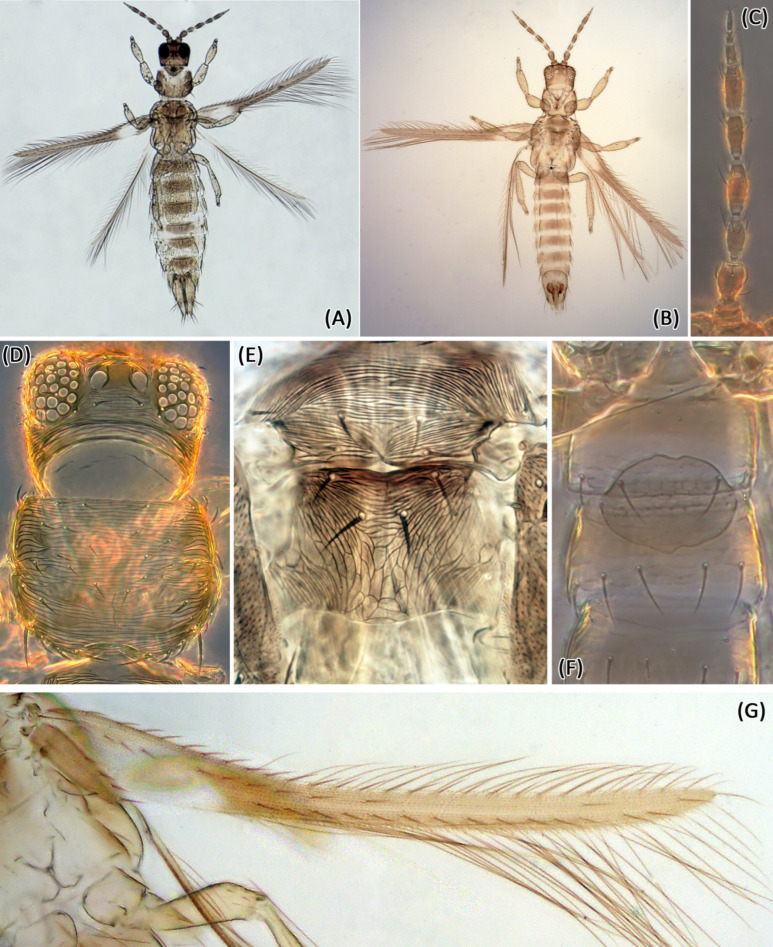
*Enneothrips flavens*. (**A**) Female; (**B**) Male; (**C**) Antenna; (**D**) Head and pronotum; (**E**) Meso- and metanotum; (**F**) Abdominal sternites II–IV (male); (**G**) Fore wing.

**Figure 5:**
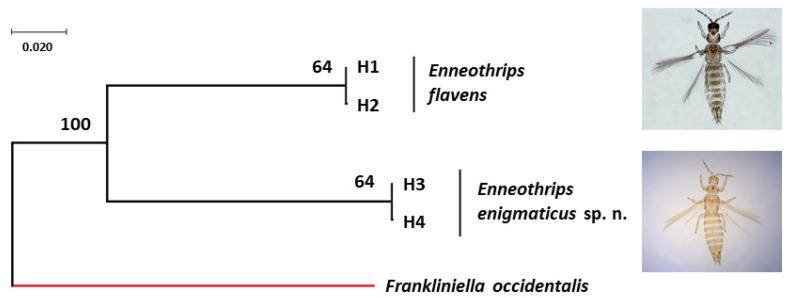
Bayesian tree of *Enneothrips flavens* and *Enneothrips enigmaticus* sp. n. haplotypes with posteriori probability values for each node/branch based on cytochrome c oxidase subunit I (*COI*) gene fragment sequences. The *COI* sequence of *Frankliniella occidentalis* (NCBI accession number: HQ930545) was used as the outgroup.

*Female macroptera*. Similar to *E. enigmaticus* sp. n. (Figure 4A,C–E,G) but with darker brown shadings, especially medially on abdominal tergites (Figure 4A), mesonotum with around 35 lines of sculpture, and reticles on metanotum with internal markings (Figure 5E).

*Male macroptera*. Similar to female (Figure 4B) but smaller and with a broad oval pore plate between the abdominal sternites II and III (Figure 4F).

Measurements (male in microns): Body length 1250. Head length 80; width 115; ocellar setae III, length 13. Pronotum length 105; width 130; posteroangular setae length 43. Fore wing length 570. Pore plate length 48; width 65. Antennal segments I–IX length 20, 30, 38, 48, 40, 38, 12, 10, 15.

*Larva II*. Similar to *Enneothrips enigmaticus* sp. n. (Figure 3G–J) Dorsal setae usually longer than in *E. enigmaticus* sp. n. (Figure 3H–J), but the measurements of specimens of both species overlap.

Measurements (in microns). Body length 1100. Head length 85; width 80; D1 setae length 18; D4 setae length 22. Pronotum length 85; width 150; D5 setae length 18; D6 setae length 28. Abdominal tergites IX length 40; width 50; setae D1 length 28; setae D2 length 30; X length 35; width 28. Antennal segments I–VII length 12, 25, 38, 42, 10, 8, 15.

Material studied. BRAZIL. Holotype female. Minas Gerais, India tea foliage, 23.v.1933 (E.J. Hambleton) (CAS). Santa Catarina: Nova Teutônia, *Allophylus*, 2.xi.1949, 2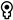 2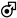 (F. Plaumann col.) (USNM); Descanso (Linha Famoso), angico (*Adenanthera macrocarpa*) 18.vii.2019, 1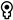 1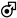 (E.F.B. Lima col.) (CHNUFPI). São Paulo: Piracicaba, shrubs, 13.iii.1997, 1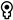 4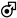 (L.A. Mound col.) (ESALQ); Santo Antonio do Pinhal, *Camponesia guazumifolia*, 21.x.2018, 5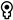 2 larvae II (E.F.B. Lima col.) (CHNUFPI). Minas Gerais: Pedralva, herbaceous Fabaceae, 15.iv.2017, 3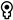 3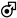, same data on *Adenanthera macrocarpa*, 1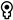; Tiradentes, Serra de São José, 16.xii.2019, 2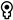 (E.F.B. Lima col.) (CHNUFPI).

Morphological comparison of specimens collected in peanuts (*E. enigmaticus* sp. n.) with the types of species currently classified in *Enneothrips* confirmed that *E. flavens* is the closest related species. However, the following morphological differences between *E. enigmaticus* sp. n. and *E. flavens* were noticed: (i) internal markings between sculpturing lines on metanotum present only in *E. flavens* (Figure 2F and Figure 4E); (ii) sculpturing lines on mesonotum less numerous in *E. enigmaticus* sp. n. (Figure 2F,G); (iii) abdominal tergites lighter and more uniformly colored in *E. enigmaticus* sp. n. (Figure 2A,B) and shaded brown medially in *E. flavens* (Figure 4A,B). In addition, individuals morphologically identical to the *E. flavens* holotype were collected in the states of São Paulo and Minas Gerais and were examined from mounted material deposited in USNM from Santa Catarina state. Among the individuals, males of *E. enigmaticus* sp. n. were brachypterous (Figure 2B), while males of *E. flavens* were always macropterous (Figure 4B).

Forward and reverse sequences were analyzed together, but as they generated different sizes, a consensus sequence was made, according to the usual procedure for estimating haplotypes. *COI* sequences were edited in a consensus size of 672 pb. Evidence of NumtS presence was not found in the sequences. The two sequences of *E. flavens* resulted in two haplotypes (H1 and H2) separated by a single mutation step (Figure 5) (NCBI accession number: H1 = MT947751 and H2 = MT947752). The seven sequences of *E. enigmaticus* sp. n. resulted also in the presence of two haplotypes (H3 and H4) separated by a single mutation step and distributed in both sample sites, Jaboticabal (H3 = 2 and H4 = 1) and Rio Branco (H3 = 1 and H4 = 3) (NCBI accession number: H3 = MT947753-MT947755 and H4 = MT947756-MT947759) (Figure 5). The intraspecific genetic distance of the *COI* gene fragment under each species was 0.0015. The interspecific genetic distance (K2P) of the *COI* gene fragment between *E. flavens* and *E. enigmaticus* sp. n. varied from 0.1872 to 0.1892.

**Figure 6:**
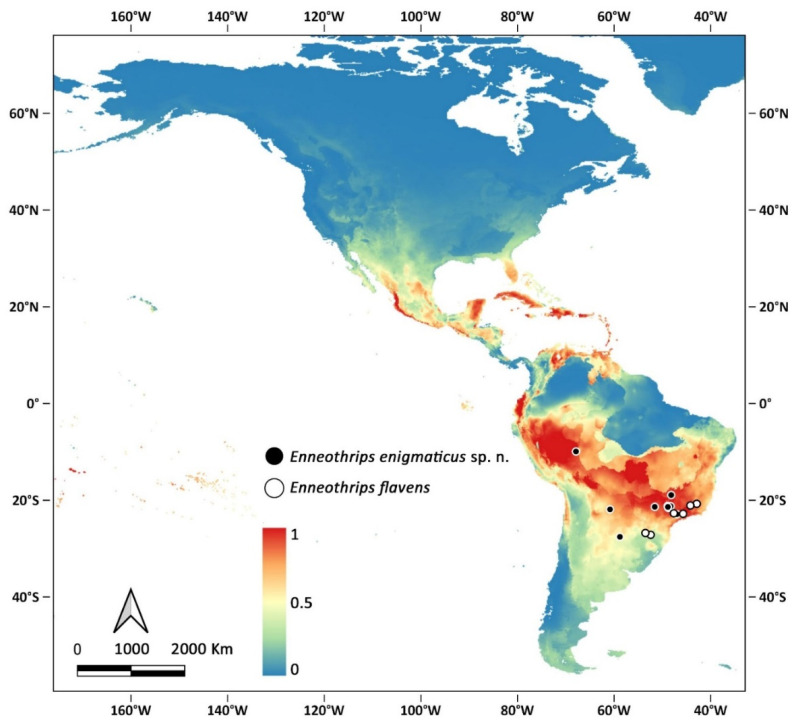
Confirmed records of *Enneothrips* spp. (circular markings) and modelling distribution of *Enneothrips enigmaticus* sp. n. in the Americas.

*Enneothrips enigmaticus* sp. n. and *E. flavens* are the only representatives of the genus occurring south of 20° S, and at present the only confirmed records are in South America, especially near the Tropic of Capricorn. Modelling, however, indicates that the species may find suitable conditions in other areas of the continent, especially in tropical countries (Figure 6).

## 4. Discussion

The differences reported above are based on comparisons of more than 100 specimens of *Enneothrips* from peanuts either collected or examined from collections and almost 30 individuals identified here as *E. flavens*, including the species holotype. Thus, they constitute robust evidence that the name historically applied to the key peanut pest has been misidentified. Males of both *Enneothrips enigmaticus* sp. n. and *E. flavens* exhibit an internal pore plate between sternites II and III, a character shared with other thrips genera from the Neotropics, namely, *Ameranathrips*, *Apterothrips*, *Baileyothrips*, *Charassothrips*, *Desartathrips*, *Enneothrips*, *Pseudothrips*, *Psydrothrips*, and *Xerothrips* [18]. The easily found males of *E. enigmaticus* sp. n. might open possibilities to study their behavior and the significance of such pore plate character. It is also possible to develop studies aiming to understand the role of the reduced wings in *E. enigmaticus* sp. n. males, a character found only in this species in the genus.

Immatures of peanut thrips have been found only from that crop, although a few adults have been collected from other plants [6]. In our surveys, we found larvae of *E. enigmaticus* sp. n. on peanuts, thus confirming the host association with the *A. hypogaea* (Figure 3K). A good number of adults have also been collected from *Arachis pintoi*¸ a possible alternative host in the same plant genus that could indicate *Arachis* spp. as hosts of *E. enigmaticus* sp. n. As for *E. flavens*, no specimens that match its holotype morphology have been collected from peanuts. On the other hand, several specimens of this thrips species were collected from at least four plant species, one of which (*Campomanesia guazumifolia*) (Figure 3L) with immatures. The large number of adult specimens from several plants and the larvae found in one of the plant species suggests that, unlike *E. enigmaticus* sp. n., *E. flavens* is probably oligophagous. That could explain the accidental collection of the species holotype from “Indian tea” plants, unrelated to *A. hypogaea*.

The genetic distance between *E. flavens* and *E. enigmaticus* sp. n. individuals is higher than that between other thrips species and supports the presence of two *Enneothrips* species [19,20,21].

Based on the resemblance and close distribution, it is feasible to indicate that *E. enigmaticus* sp. n. and *E. flavens* are two sister species. A possibility to explain the origin of the two species is an ancestor in South America whose populations were separated by a functional character. While *E. enigmaticus* sp. n. specialized breeding on *Arachis*, *E. flavens* became an oligophagous species. *Arachis hypogaea*, the main host of *E. enigmaticus* sp. n. is native to South America, most probably to the area around Southern Bolivia, Northern Argentina, and Western Paraguay [22,23], but also reaching Peru, Chile, and Western Brazil. As host-specific, that might be the area of origin of the thrips species as well. Specimens of *E. flavens* have been collected from Western Santa Catarina state, close to the border of Brazil with Paraguay and Argentina, reinforcing the idea of a shared natural occurrence (Figure 3), also evidenced by the modelling distribution of the new species and the confirmed records of *E. flavens*. Another possibility is that a lineage of *Enneothrips* invaded Central and Southern South America and was subsequently divided by vicariance. The western population would have specialized in *Arachis*, giving raise to *E. enigmaticus* sp. n., which was subsequently transported further into Southeastern Brazil with its host on cultivated peanuts. These or other explanations can be explored in a phylogeographic analysis.

## 5. Conclusions

Based on morphological, biological, and molecular evidence, we conclude that the peanut thrips, *Enneothrips enigmaticus* sp. n., was previously misidentified as *Enneothrips flavens* and is here described as a new species.

## Figures and Tables

**Table 1:** Approximate coordinates of reliable available records of *Enneothrips enigmaticus* sp. n. and *Enneothrips flavens* in South America.

***Enneothrips enigmaticus* sp. n. **			
**Country**	**Locality**	**Approximate Coordinates**	**Source**
Argentina	Corrientes	27°35′02.00″ S; 58°47′31.00″ W	MLP
Brazil	Rio Branco, AC	9°55′43.24″ S; 67°53′54.80″ W	CHNUFPI
	Santa Adélia, SP	21°23′58.00″ S; 48°51′42.00″ W	CHNUFPI
	Jaboticabal, SP	21°14′23.40″ S; 48°17′45.22″ W	ESALQ/CHNUFPI
	Tupi Paulista, SP	21°22′59.00″ S; 51°34′32.00″ W	ESALQ
	Campinas, SP	22°54′25.00″ S; 47°00′53.00″ W	ESALQ
	Pindorama, SP	21°11′11.00″ S; 48°54′19.00″ W	CHNUFPI
	Itápolis, SP	21°35′19.00″ S; 48°46′36.00″ W	CHNUFPI
	Uberlândia, MG	18°56′03.00″ S; 48°10′38.00″ W	CHNUFPI
	Paraná	-	MLP
Paraguay	Boquerón	21°54′04.00″ S; 60°49′59.00″ W	CHNUFPI
***Enneothrips flavens***			
**Country**	**Locality**	**Approximate Coordinates**	**Source**
Brazil	Minas Gerais *	20°44′51.00″ S; 42°53′00.00″ W	CAS
	Pedralva, MG	22°15′55.00″ S; 45°24′37.00″ W	CHNUFPI
	Tiradentes, MG	21°05′56.84″ S; 44°12′12.00″ W	CHNUFPI
	Santo Antonio do Pinhal, SP	22°48′38.55″ S; 45°42′14.24″ W	CHNUFPI
	Piracicaba, SP	22°42′48.00″ S; 47°37′39.77″ W	ESALQ
	Nova Teutônia, Seara, SC	27°9′46.00″ S; 52°25′28.00″ W	USNM
	Descanso, SC	26°47′2.00″ S; 53°30′38.00″ W	CHNUFPI

* Possibly in the municipality of Viçosa.
